# Efficacy of Posaconazole Prophylaxis for Fungal Disease in Hematology Patients Treated With Chemotherapy and Transplantation: An Open-Label, Prospective, Observational Study

**DOI:** 10.3389/fmicb.2020.00349

**Published:** 2020-03-19

**Authors:** Weiyang Li, Fan Xia, Haixia Zhou, Huiying Qiu, Depei Wu, Xiao Ma, Aining Sun

**Affiliations:** ^1^Department of Hematology, Jiangsu Institute of Hematology, National Clinical Research Center for Hematologic Diseases, The First Affiliated Hospital of Soochow University, Suzhou, China; ^2^Department of Clinical Pharmacology, The First Affiliated Hospital of Soochow University, Suzhou, China

**Keywords:** invasive fungal infection, posaconazole, serum drug concentration, antifungal prophylaxis, Chinese hematology patients

## Abstract

**Background:**

Posaconazole (PCZ) is used prophylactically to prevent invasive fungal infections (IFIs) in patients with hematological malignancies.

**Objective:**

To evaluate the cut-off serum concentration of PCZ for successful IFI prophylaxis in Chinese subjects.

**Patients and Methods:**

A total of 74 patients treated with induction chemotherapy (*n* = 10) and allogeneic hematopoietic stem cell transplantation (HSCT) (*n* = 64), who received PCZ prophylactically as an oral suspension for >7 days, were included in the study. Clinical, radiological, microbiological culture results, and treatment responses were analyzed and drug concentration assays performed.

**Results:**

The overall incidence of possible, probable, and proven IFIs was 13.5% (10/74), with five patients in the chemotherapy group and five in the HSCT group. The PCZ serum concentration in most patients (54/63) was in the range of 0.25–1.0 μg/ml, and this concentration range was significantly associated with the success rate of PCZ prophylaxis. A cut-off value of 0.47 μg/ml can be considered as an evaluation index for PCZ prophylaxis. Taking a proton pump inhibitor (PPI) would reduce the PCZ blood concentration, but not affect the IFD breakthrough point. PCZ treatment for hematopoietic malignancy or HSCT patients with a serum concentration of PCZ < 0.47 μg/ml were risk factors for PCZ prophylaxis of IFIs, determined by univariable and multivariable regression analyses.

**Conclusion:**

The serum concentration of PCZ was related to the incidence of IFIs and a serum concentration of >0.47 μg/ml is highly recommended to avoid IFIs after chemotherapy or HSCT.

**Clinical Trial Registration:**

Chinese Clinical Trial Registry: ChiCTR1900026294.

## Introduction

Invasive fungal infections (IFIs) are a common complication of chemotherapy and transplantation in immunocompromised patients. *Candida* and *Aspergillus* species are the most common causes of IFIs, but other yeasts and filamentous fungi including *Fusarium*, *Scedosporium*, and *Purpureocillum* are emerging pathogens (Gullo, 2009). Given the limited clinical manifestations and the risk of developing multiresistant strains, the methodology for early diagnosis and treatment of IFI is very limited (Casucci et al., 2014). Once diagnosed, early initiation of appropriate antifungal therapy is essential for reducing the extremely high morbidity and mortality rates (Gullo, 2009). Since the early 1950s, amphotericin B deoxycholate has been the first choice of drug to treat IFIs (Fluckiger et al., 2006), but several alternative treatment options have emerged in recent years. Lipid-based formulations of amphotericin B and expanded-spectrum triazoles (i.e., voriconazole and posaconazole) and echinocandins have been introduced in clinical practice for both the prevention and treatment of IFIs (Pound et al., 2011). Breakthrough of fungal infections and treatment failures may well be associated with low serum concentration of these drugs (Lerolle et al., 2014; Kim et al., 2017). In contrast, high serum concentration of these drugs are associated with toxic effects (Andes et al., 2009).

Posaconazole (PCZ) is an extended-spectrum triazole with broad activity against a variety of fungal pathogens, including yeasts and molds (Mehta and Langston, 2009). PCZ has been used for the prophylaxis of IFI in patients undergoing chemotherapy or HSCT (Leclerc et al., 2018). Previous studies have reported a significant association between a steady-state lower serum concentration of PCZ and a higher breakthrough rate of IFIs (Dolton et al., 2012; Moore et al., 2015; Chin et al., 2017). The absorption of PCZ is significantly increased when administered with a (high-fat) meal (Krishna et al., 2009). PCZ is not metabolized by CYP450 isoenzymes and is mostly excreted unchanged in the feces (Spencer et al., 2011). Between 20 and 30% of the PCZ dose is glucuronidated by the phase II enzyme, UGT1A4. Therefore, inhibitors or inducers of this elimination pathway may affect the serum concentration of PCZ (Dolton et al., 2012). With unchanged administration of phenytoin (to prevent seizures), on the 10th day of a steady-state serum concentration, the PCZ level was reduced by as much as 50%, which corresponds to a 90% increase in the steady-state PCZ clearance rate (Leung et al., 2015). At present, routine measurements of the serum concentration of PCZ is not carried out in clinical practice in China.

Proton pump inhibitors (PPIs) are widely used for the treatment of peptic ulcers and gastrointestinal hemorrhage and are often co-administered with antifungal agents in patients at a high risk of contracting IFIs (Yan et al., 2018). However, PPIs are likely to inhibit the metabolism of PCZ and thus decrease the serum concentration (Chayakulkeeree et al., 2015; Yan et al., 2018). Therefore, in the present study, the effect of a PPI on the serum concentration of PCZ was investigated. It is noteworthy that PCZ has a long half-life of approximately 35 h and the steady-state concentration is achieved after 7 days of administration. There is minimum fluctuation around the mean values once the steady-state concentration is reached (Chae et al., 2015). Hence, the serum concentration on day 7 was regarded as the average steady-state PCZ serum concentration for each subject. A study of 4,192 Chinese patients in 2015 demonstrated that antifungal prophylaxis produced an independent protective effect, but that this therapy was not commonly used, even in high-risk patients (Sun et al., 2015). PCZ showed good efficacy as a prophylaxis agent against IFIs in high-risk neutropenic Chinese patients and was well tolerated during long-term use (Huang et al., 2012; Shen et al., 2013). It is imperative to establish a definitive cut-off value to provide guidance for PCZ administration and thus improve its prophylaxis efficacy.

Therefore, we explored the relationship between the serum concentration of PCZ and its prophylaxis action, to determine whether there are predictable risk factors for prophylaxis therapy failure, to provide unequivocal evidence for a reduced breakthrough rate for IFIs and to promote diagnosis of fungal infections in patients with neutropenia or those who are immunocompromised.

## Participants and Methods

### Participants

One hundred sixty participants with blood disease who had been treated with PCZ for antifungal prophylaxis were screened between March 1, 2017 and January 30, 2019 in the First Affiliated Hospital of Suzhou University. A total of 74 subjects were enrolled in the study according to the inclusion criteria. They received 5 ml of PCZ oral solution three times per day for 0.5–1 month and were closely monitored. In addition to using PCZ to prevent fungal infections, we also administered PPIs (omeprazole, lansoprazole, or pantoprazole) to study subjects with obvious gastrointestinal reactions or a past history of gastrointestinal disease in an attempt to protect their gastrointestinal mucosa.

During the dose maintenance period, if a participant needed to increase the dose or use other antifungal drugs due to the onset of an IFI, they were instructed to immediately contact the researcher to record the change in the additional drugs or dose administered and to indicate the reasons for their change in medication. The medical ethics committee of the First Affiliated Hospital of Suzhou University approved the study (2015 IRB119).

### Inclusion and Exclusion Criteria

Hematology patients ≥13 years old, who underwent HSCT transplantation or induction chemotherapy and were prescribed PCZ, were enrolled. Blood diseases included aplastic anemia, acute myeloid leukemia, acute lymphocytic leukemia, non-Hodgkin’s lymphoma, multiple myeloma, and others. Those subjects treated with induction chemotherapy were expected to have, or had documented, persistent neutropenia [absolute neutrophil count (ANC) ≤ 0.5 × 10^9^/L] sustained for at least 7 days, were also enrolled. Subjects who were allergic to PCZ, were <13 years of age, without agranulocytosis after induction chemotherapy, and whose drug concentrations were not detected at the prescribed intervals were excluded. In addition, subjects for economic grounds, death, or other reasons who failed to complete the course of treatment (0.5–1 months) of PCZ were also excluded from analyses. The patient or their authorized guardian provided signed informed consent permitting them to participate in the clinical trial. Any predictable factors that may have increased patient risk or interfered with the clinical trial results were also considered to be exclusion criteria.

### Experimental Design

This was an open, prospective, observational single-center study. Medical information generated during routine clinical diagnosis and treatment was documented. Blood samples (2 ml) were collected from most participants at 7-, 14-, and 21-day intervals after treatment with PCZ. A serum sample, collected 30 min before the first dose of the drug was taken in the morning (usually carried out during other routine clinical blood tests), was used to measure the drug concentration. One week later, a follow-up visit was scheduled either in person or by telephone to collect the participants’ personal medical information, analyze the possible causes of any breakthrough IFIs, and to securely store the data.

### Measurement of Drug Concentrations

An ACQUITY ultra high-performance liquid chromatograph equipped with Xevo TQS triple four-bar tandem mass spectrometer and positive ion mode multireaction monitoring system (UPLC-MS/MS; Waters, United States) was used to determine the PCZ concentration in serum using posaconazole-d4 as the internal standard. Protein precipitation pretreatment was employed as the sample preparation. The analytical column was an ACQUITY UPLC BEH C18 (100 mm × 2.1 mm, 1.7 μm). The mobile phase consisted of acetonitrile and 0.04% formic acid solution (50:50). The flow rate was set at 0.3 ml/min. The specificity, standard curve, lower limit of quantitation, precision, recovery rate, matrix effect, and stability were all evaluated.

### Primary and Secondary Endpoints

#### Primary Endpoints

The primary endpoints included the improvement or progression of disease and the breakthrough rate of IFIs after PCZ administration. Treatment failure was defined as a possible, probable, or proven infection according to EORTC criteria (De Pauw et al., 2008).

#### Secondary Endpoints

The secondary endpoints were changes in the PCZ serum concentration and the neutrophil count before and after treatment.

#### Adverse Events

Adverse events (AEs) were considered to be any harmful or unforeseeable medical events that occurred when, or after, a subject had taken any dose of PCZ, but they did not necessarily have a causal relationship with the drug. Thus, an AE can be any unexpected symptom, sign, or condition (including clinically significant laboratory abnormalities) that may be associated with the use of the drug in question, regardless of whether it is drug-related or not. Serious AEs are those that occur after taking any dose and must be fatal, endanger life, require acute or prolonged hospitalization, induced disability, trigger cancer. Based on appropriate medical judgment, those events that did not result in death, life-threatening symptoms, or hospitalization were considered to be significant AEs that may have harmed the subject and required medical or surgical intervention (Raad et al., 2006).

#### Sample Size

According to the IFD breakthrough rate of 23% after fungal prophylaxis, the target value of the single group was set at 10%, the significance level at 0.05, and the test efficacy at 80%. A sample size of 48 cases was needed. Assuming a follow-up loss rate of 20%, a total of 60 participants were required.

#### Statistical Analysis

Data in this study are expressed as the mean ± standard deviation (SD). All statistical analyses were performed using GraphPad Prism version 6 software (GraphPad Software, La Jolla, CA, United States). Comparisons of two groups with normally distributed data were analyzed using Student’s *t*-test. Comparisons among multiple groups were performed by analysis of variance (one-way ANOVA or two-way ANOVA) followed by Bonferroni’s *post hoc* test. All risk factors related to IFI failure and demographic characteristic variables were calculated and analyzed with a univariate logistic regression model. Only significant factors analyzed by univariate logistic regression analysis were included for multivariate logistic regression analysis with adjustment or without adjustment for various confounding factors (age, gender, and ECOG scores). The odds ratios and 95% confidence intervals (CIs) in both the univariate and multivariate logistic regression models were used to quantify relationships between each variable and the outcome event (IFI failure). A value of *P* < 0.05 was considered to be statistically significant.

## Results

### Demographic Data and Characteristics of the Enrolled Patients

**FIGURE 1:**
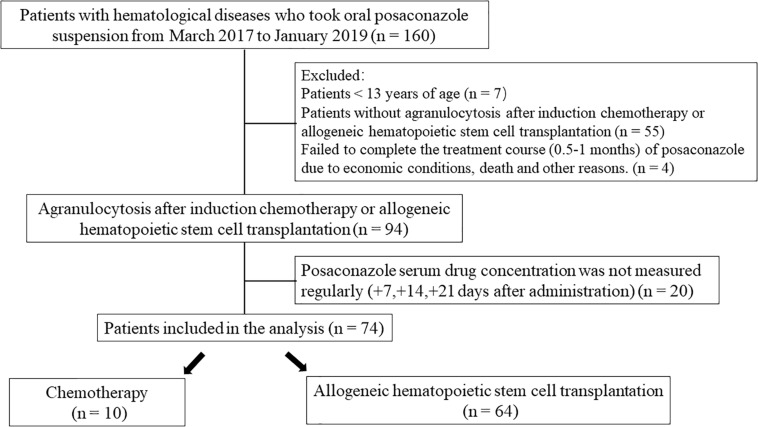
Flow chart of the study.

A total of 74 patients were enrolled in the study and comprised 48 males (64.9%) and 26 females (35.1%) (Figure 1).

Sixty patients (81.1%) were characterized as Eastern Cooperative Oncology Group Performance Status (ECOG) 1 level. Patients were divided into either a transplant group (64 patients, 86.5%) or a chemotherapy group (10 patients, 13.5%). The average neutrophil count was 4.2 ± 4.3 × 10^9^/L (Table 1).

**TABLE 1:** Characteristics of the participants.

	**Number of participants (percentage)**
**Gender**	
Male	48 (64.9)
Female	26 (35.1)
Age (years)	32.7 ± 13.8
**ECOG**	
1	60 (81.1)
2	11 (14.9)
3	3 (4.0)
**Therapeutic methods**	
Chemotherapy	10 (13.5)
AML	3 (4.1)
ALL	7 (9.5)
HSCT	64 (86.5)
Haploidentical transplantation	43 (58.1)
Total compatibility transplantation	21 (28.4)
GVHD	20(31.3)
Neutrophil (× 10^9^/L)	4.2 ± 4.3
**Chemotherapy participants**	
Alanine transaminase (ALT, U/L)	21.7 ± 10.1
Aspartate transaminase (AST, U/L)	32.4 ± 16.3
Total bilirubin (TBIL, μmol/L)	10.6 ± 5.5
Blood urea nitrogen (BUN, mmol/L)	4.2 ± 1.6
Creatinine (Cr, μmoI/L)	58.7 ± 13.7

*GVHD, graft-versus-host disease.*

### Correlation Between Failure to Prevent IFIs and the Serum Concentration of PCZ

**FIGURE 2:**
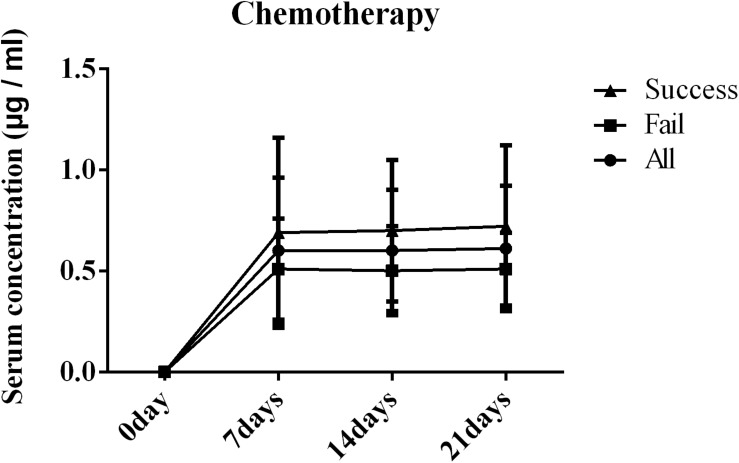
Blood concentration of posaconazole (PCZ) on days 7, 14, and 21 after administration in patients with blood disease or who received a hematopoietic stem cell transplantation (HSCT).

The serum concentrations of PCZ on days 7, 14, and 21 after administration are shown in Figure 2. The results clearly showed significant differences in the serum concentration of PCZ between success and failure patients (*P* = 0.000, *P* < 0.000, *P* = 0.002). There was no statistical difference between successful patients and the overall study population.

In order to clarify whether the failure to prevent IFDs was due to an inappropriate serum concentration of PCZ or the treatment mode of blood diseases (chemotherapy and transplantation), a correlation analysis was performed (Table 2). The results revealed that the failure of PCZ to prevent IFIs at any time was related to the patients’ serum PCZ concentration (*P* = 0.018, *P* = 0.009, and *P* = 0.048) and was not correlated with the treatment method (*P* = 0.348, *P* = 0.262, and *P* = 0.527). Therefore, more attention should be paid to the serum drug concentration in PCZ prophylaxis patients.

**TABLE 2:** Correlation analysis of blood drug concentration, success rate, and treatment mode on days 7, 14, and 21 of treatment.

	**Blood drug concentration (μg/ml)**					
	**Day 7**		**Day 14**		**Day 21**	
	**F**	***P-*value**	**F**	***P-*value**	**F**	***P-*value**
Model	3.38	0.040	4.29	0.017	2.24	0.11
Success/failure	5.86	0.018	7.31	0.009	4.08	0.048
Chemotherapy/transplantation	0.89	0.348	1.28	0.262	0.41	0.527

We further analyzed the number and proportion of participants with successful or failed fungal infection prophylaxis and their serum drug concentration ranges. The results revealed that there was no prophylaxis failure in any subject in which the serum concentrations were >0.75 μg/ml. The serum concentrations in the majority of the subjects were in the range 0.26–0.50 μg/ml (27/74, 36.5%) with 17.4% failure and 0.51–0.75 μg/ml (23/74, 36.5%) with 9.5% failure, whereas in the range 0.0–0.25 μg/ml, the failure rate was (4/7) 57.1%. Of 10 subjects in whom PCZ prophylaxis failed, five cases belonged to the chemotherapy group (5/10, 50%) and five to the transplant group (5/64, 7.8%), with serum concentrations ranging between 0.25–0.75 and 0.25–0.5 μg/ml, respectively. Therefore, we suggest that the concentration of PCZ in chemotherapy patients should be >0.76 μg/ml and in transplant patients >0.50 μg/ml, concentrations which are likely to produce satisfactory prophylaxis (Table 3). In addition, the cut-off concentration value was calculated to be 0.47 μg/ml for success or failure of PCZ prophylaxis for the entire study population.

**TABLE 3:** Success rate of PCZ prophylaxis for different drug concentrations.

**Blood drug concentration (μg/ml) (D7)**	**Total**		**Chemotherapy**	**Transplant**
	**Success/total**	**Failure (%)**	**Failure (%)**	**Failure (%)**
0.0–0.25	3/7 (42.9)	4 (57.1)	1 (50.0)	3 (60.0)
0.26–0.50	19/23 (82.6)	4 (17.4)	2 (66.7)	2 (10.0)
0.51–0.75	19/21 (90.5)	2 (9.5)	2 (66.7)	0 (0)
0.76–1.00	6/6 (100)	0 (0)	0 (0)	0 (0)
1.00–1.25	6/6 (100)	0 (0)	–	0 (0)
1.25–1.50	3/3 (100)	0 (0)	0 (0)	0 (0)
1.51–1.75	3/3 (100)	0 (0)	–	0 (0)
1.76–2.00	2/2 (100)	0 (0)	–	0 (0)
2.01–2.25	–	–	–	–
2.26–2.50	3/3 (100)	0 (0)	–	0 (0)
Total successful/failure cases	64	10	5	5

The steady-state threshold range of the PCZ concentration in 56.2–68.8% of male subjects was 0.26–0.75 μg/ml and in 61.5–75.0% of female subjects 0.26–1.00 μg/ml. The threshold range of the steady-state PCZ concentration in 60–70% of chemotherapy patients was 0.00–0.50 μg/ml and in 30–38% of transplant patients 0.26–0.75 μg/ml (Supplementary Table 1). Therefore, in order to reduce the breakthrough rate of IFIs, increasing the serum PCZ concentration above the cut-off value of 0.47 μg/ml will be necessary.

### Adverse Reactions After PCZ Prophylaxis

After 2 weeks of PCZ prophylaxis, except for a significant decrease in creatinine levels, other indexes including alanine transaminase (ALT), aspartate transaminase (AST), total bilirubin (TBIL), and blood urea nitrogen (BUN) were not significantly changed before or after treatment. These findings indicated that there was no significant inference of PCZ with liver and kidney functions (Table 4).

**TABLE 4:** Changes in liver and kidney function indexes before and after PCZ prophylaxis.

	**Premedication concentration**	**Concentration after 2 weeks**	***P*-value**
Alanine transaminase (ALT, U/L)	59.8 ± 89.8	43.6 ± 47	0.15
Aspartate transaminase (AST, U/L)	42.6 ± 53.3	31.5 ± 19.8	0.16
Total bilirubin (TBIL, μmol/L)	14.7 ± 24.0	15.9 ± 23.0	0.23
Blood urea nitrogen (BUN, mmol/L)	5.4 ± 3.1	5.3 ± 2.6	0.92
Creatinine (Cr, μmoI/L)	62.2 ± 25.3	55.4 ± 19.7	0.000

### Effect of PPIs on PCZ Prophylaxis

The results shown in Table 5 revealed that PPI therapy did not influence the success rate of PCZ prophylaxis (*P* = 0.527). It actually reduced the serum concentrations of PCZ in subjects after 1, 2, and 3 weeks of therapy (all *P* < 0.05) (Table 5), which implied that PPIs reduced the serum levels of PCZ, but they still remained higher than the cut-off value of 0.47 μg/ml. This is the likely reason why there was no reduction in the incidence of IFIs during PCZ prophylaxis.

**TABLE 5:** The effect of PPI drug on the success rate of PCZ prophylaxis and blood concentration of PCZ.

	**Participants (n,%)**		**Serum concentration of PCZ**		
	**PCZ prophylaxis success**	**PCZ prophylaxis failure**	**Duration (Days)**		
			**D7**	**D14**	**D21**
Total	64 (86.50)	10 (13.50)	–	–	–
Without taking PPI	19 (95.00)	2 (9.50)	0.70 (0.44–1.32)	0.83 (0.56–1.19)	0.78 (0.56–1.20)
Taking PPI	45 (84.90)	8(15.10)	0.55 (0.39–0.75)	0.56 (0.42–0.81)	0.52 (0.38–0.73)
*P*-value	0.527		0.036	0.042	0.041

### Multiple Regression Analysis of Factors Affecting IFI Breakthrough Rate

Finally, the factors on IFI breakthrough rate were evaluated by univariable and multivariable regression analyses. The analyses revealed that chemotherapy was a highly significant factor leading to IFI breakthrough [OR 95% CI: 15.46 (2.48–96.40)]. In addition, a blood concentration of PCZ < 0.47 μg/ml is also a risk factor [OR 95% CI: 0.10 (0.02–0.67)] for the IFI incidence. Both factors remained significant after adjustment to age, gender, and ECOG levels (Table 6).

**TABLE 6:** Multiple regression analysis of factors affecting the IFI breakthrough rate.

	**Univariable**			**Multivariable (before adjusting age, gender and ECOG)**		**Multivariable (after adjusting age, gender and ECOG)**	
		**OR 95% CI**	***P*-value**	**OR 95% CI**	***P*-value**	**OR 95% CI**	***P*-value**
**Variable**							
Gender	Female	1.0	–	–	–	–	–
	Male	0.49 (0.13–1.87)	0.296	–	–	–	–
Years		1.03 (0.98–1.08)	0.195	–	–	–	–
ECOG	1	1.0	–	–	–	–	–
	≥2	0.44 (0.05–3.76)	0.450	–	–	–	–
Therapeutic method	Transplant	1.0	–	1.0	–	1.0	–
	Chemotherapy	11.80 (2.53–55.01)	0.002	15.46 (2.48–96.40)	0.003	14.66 (2.05–104.95)	0.008
Transplant type	Total compatibility transplantation	1.0	–	–	–	–	–
	Haploidentical transplantation	>999.9 (<0.001–>999.999)	0.953	–	–	–	–
GVHD	0	1.0	–	–	–	–	–
	1	0.70 (0.07–7.20)	0.766	–	–	–	–
**PCZ prophylaxis**							
Neutrophil count	–	0.98 (0.83–1.15)	0.775	–	–	–	–
ALT (U/L)	–	0.96 (0.91–1.02)	0.166	–	–	–	–
AST (U/L)	–	0.99 (0.95–1.02)	0.364	–	–	–	–
BIL (μmol/L)	–	0.94 (0.81–1.09)	0.431	–	–	–	–
BUN (mmol/L)	–	0.93 (0.72–1.19)	0.546	–	–	–	–
Cr (μmol/L)	–	1.00 (0.97–1.03)	0.965	–	–	–	–
Drug therapy	PCZ alone	1.0	–	–	–	–	–
	Combined with PPI drugs	23.75 (2.15–262.42)	0.010	–	–	–	–
	Combined with other drugs	1.85 (0.19–17.73)	0.592	–	–	–	–
D7 drug blood concentration	<0.60	1.0	–	–	–	–	–
	≥0.60	0.21 (0.04–1.05)	0.058	–	–	–	–
D7 drug blood concentration	≤0.47	1.0	–	1.0	–	1.0	–
	>0.47	0.13 (0.03–0.67)	0.015	0.10 (0.02–0.67)	0.017	0.07 (0.01–0.61)	0.017

## Discussion

The present study explored the correlation between the serum concentration of PCZ and the breakthrough rate of IFIs in hematology patients who received chemotherapy or allogeneic HDCTs. The data presented is based on 74 enrolled patients who underwent PCZ prophylaxis. The results revealed that 10 patients (13.5%) in total had progression of fungal infections including five patients who received chemotherapy and five who underwent transplantation, which is in good agreement with previously published data. For example, a study in the United States reported that 15% of patients who received PCZ tablets for prophylaxis had breakthrough IFIs (Pagano and Mayor, 2018). However, a single-center study of patients with acute myeloid leukemia documented four breakthrough IFIs (4/84, 4.8%) in the PCZ group, which was lower than the normal range (Ozkocaman et al., 2018). In addition, different formulations of PCZ may be related to the breakthrough rate of IFIs, since one study noted that *Aspergillus* infections occurred in nine (8.7%) patients in the PCZ suspension group and in no patient who took the tablet formulation (Leclerc et al., 2018). Other antibiotic drugs were evaluated with regard to prophylaxis efficacy and showed similar rates. A 12% breakthrough rate of IFIs was found in hematologic malignancy patients prescribed isavuconazole for prophylaxis (DeVoe et al., 2018). Invasive *Aspergillus* breakthrough occurred in 6/46 (13%) of patients treated with caspofungin, with symptoms that included persistent fever and neutropenia (Lafaurie et al., 2010). Overall, our study has demonstrated a similar prophylaxis efficacy of PCZ, but it is still unacceptably low and needs to be considerably improved.

In the present study, a cut-off value of 0.47 μg/ml has been proposed, which provides a critical evaluating index during PCZ prophylaxis. Given the high risk of mortality from IFIs and inter- and intra-subject variability in PCZ pharmacokinetics, therapeutic drug monitoring (TDM) should be a strategy that should be employed to optimize drug therapy (Yi et al., 2017). A serum level of PCZ > 0.70 μg/ml is normally the target concentration for effective prophylaxis (Maleki et al., 2018). A meta-analysis of 28 studies that involved 1,930 patients found that patients with PCZ serum concentrations >0.5 mg/L were twice as likely to achieve successful responses compared to those with lower concentrations (Chen et al., 2018). In our study, a cut-off concentration value of 0.47 μg/ml is recommended, which provides a critical evaluating index for PCZ prophylaxis during the TDM process in China.

In addition, our results showed that the breakthrough point in male subjects was 10.4% and in female subjects 19.2%, which implies that gender may significantly influence drug action: males (median = 521.50 ng/ml) exhibited greater PCZ serum concentrations than females (median = 376.50 ng/ml, *P* = 0.028) among the 172 patients who contracted IFIs (Allegra et al., 2017). The study subjects’ ages, body mass index (BMI), and the administered PCZ dose did not significantly affect PCZ pharmacokinetics (Allegra et al., 2017). However, a study that evaluated the risk factors for sub-therapeutic levels of PCZ tablets found that male gender was one factor associated with PCZ troughs (<0.7 μg/ml) (Tang et al., 2017). Our data showed that the breakthrough rate in male subjects (10.4%) was lower than for female subjects (19.2%). Further investigation with a larger cohort of subjects will be required before an unequivocal conclusion can be reached.

Proton pump inhibitors are among the most frequent drugs administered to hematology patients (Neubauer et al., 2010). Patients who are likely to require antifungal therapy often have abnormal gastric pH levels and present with gastrointestinal disorders, which are treated with PPIs (Krishna et al., 2009). Patients who receive PPIs are more likely to have lower concentrations of PCZ in their serum (Cojutti et al., 2013; Tang et al., 2017), a finding in agreement with the present study (Table 5). However, multivariable regression analysis showed that the administration of PPIs had a limited influence on the failure rate of prophylactic antifungal treatment (Table 6). One possible reason is that PPIs can effectively prevent gastrointestinal mucosal inflammation elicited by chemotherapy or transplantation, and a complete mucosal barrier can reduce the chance of acquiring fungal infections. Additionally, the median PCZ serum concentration in PPI combined with PCZ-treated subjects was above the cut-off value (0.47 μg/ml), which may also explain the non-significant difference in the breakthrough rate between the PPI and non-PPI-treated groups.

Apart from the small number of subjects in the chemotherapy group, the limitations of the present study were its single-center design with limited generalizability, and there may also have been selection biases that affected the extrapolation of research results. However, our study used continuous enrollment to reduce selection biases to some extent. In addition, it is fair to say that confounding biases are an unavoidable problem in observational research, but a multifactorial model was used to analyze the influencing factors for fungal infection prophylaxis failure in order to control these.

## Conclusion

In conclusion, we established a cut-off concentration value of PCZ in Chinese hematology patients treated with chemotherapy and HSCT who were at a significant risk of IFIs. We found that PPI medication decreased the PCZ serum concentration when PCZ prophylaxis was combined with PPI treatment. However, the risk of onset of IFIs occurred only when the serum blood concentration was <0.47 μg/ml. It is strongly recommended that PCZ serum concentrations should be monitored, especially in patients at a high risk of contracting IFIs.

## Data Availability Statement

The datasets generated for this study are available on request to the corresponding author.

## Ethics Statement

The studies involving human participants were reviewed and approved by the Medical Ethics Committee of the First Affiliated Hospital of Suzhou University approved the study (2015 IRB119). Written informed consent to participate in this study was provided by the participants’ legal guardian/next of kin.

## Author Contributions

WL and FX conceptualized the study. HQ carried out the analysis and interpretation. WL and HZ contributed to the data collection and analysis. WL wrote the original draft. HZ, DW, XM, and AS wrote, reviewed and edited the manuscript. All authors read and approved the submitted manuscript.

## Conflict of Interest

The authors declare that the research was conducted in the absence of any commercial or financial relationships that could be construed as a potential conflict of interest.
