# Supplementary material for: Efficacy of Posaconazole Prophylaxis for Fungal Disease in Hematology Patients Treated With Chemotherapy and Transplantation: An Open-Label, Prospective, Observational Study
Source: Front Microbiol. 2020 Mar 19;11:349. doi: 10.3389/fmicb.2020.00349 (PMC7096356; doi:10.3389/fmicb.2020.00349)
Supplement: Supplementary file 1 [file Table_1.DOCX]

**Supplementary Table 1: Threshold range for steady-state posaconazole concentration**

| Blood drug concentration | Total cases | | | Male | | | Female | | | Chemotherapy | | | Transplant | | |
| --- | --- | --- | --- | --- | --- | --- | --- | --- | --- | --- | --- | --- | --- | --- | --- |
|  | 7 | 14 | 21 | 7 | 14 | 21 | 7 | 14 | 21 | 7 | 14 | 21 | 7 | 14 | 21 |
| 0.0 – 0.25 | 7 (9.5) | 6 (8.1) | 6 (9.8) | 2 (20) | 1 (10) | 1 (10) | 5 (7.8) | 5 (7.8) | 5 (9.8) | 2 (20) | 1 (10) | 1 (10) | 5 (7.8) | 5 (7.8) | 5 (9.8) |
| 0.26 – 0.50 | 23 (31.1) | 18 (24.3) | 17 (27.9) | 3 (30) | 2 (20) | 3 (30) | 20 (31.3) | 16 (25) | 14 (27.5) | 3 (30) | 2 (20) | 3 (30) | 20 (31.3) | 16 (25) | 14 (27.5) |
| 0.51 – 0.75 | 21 (28.4) | 21 (28.4) | 20 (32.8) | 3 (30) | 4 (40) | 4 (40) | 18 (28.1) | 17 (26.6) | 16 (31.4) | 3 (30) | 4 (40) | 4 (40) | 18 (28.1) | 17 (26.6) | 16 (31.4) |
| 0.76 – 1.00 | 6 (8.1) | 14 (18.9) | 7 (11.5) | 1 (10) | 2 (20) | 1 (10) | 5 (7.8) | 12 (18.8) | 6 (11.8) | 1 (10) | 2 (20) | 1 (10) | 5 (7.8) | 12 (18.8) | 6 (11.8) |
| 1.00 – 1.25 | 6 (8.1) | 7 (9.5) | 3 (4.9) |  | 1 (10) |  | 6 (9.4) | 6 (9.4) | 3 (5.9) |  | 1 (10) |  | 6 (9.4) | 6 (9.4) | 3 (5.9) |
| 1.25 – 1.50 | 3 (4.1) | 1 (1.4) | 4 (6.6) | 1 (10) |  | 1 (10) | 2 (3.1) | 1 (1.6) | 3 (5.9) | 1 (10) |  | 1 (10) | 2 (3.1) | 1 (1.6) | 3 (5.9) |
| 1.51 – 1.75 | 3 (4.1) | 2 (2.7) | 2 (3.3) |  |  |  | 3 (4.7) | 2 (3.1) | 2 (3.9) |  |  |  | 3 (4.7) | 2 (3.1) | 2 (3.9) |
| 1.76 – 2.00 | 2 (2.7) | 2 (2.7) | 1 (1.6) |  |  |  | 2 (3.1) | 2 (3.1) | 1 (2) |  |  |  | 2 (3.1) | 2 (3.1) | 1 (2) |
| 2.01 – 2.25 |  | 1 (1.4) | 1 (1.6) |  |  |  |  | 1 (3.9) | 1 (5) |  |  |  |  |  |  |
| 2.26 – 2.50 | 3 (4.1) | 2 (2.7) |  | 2 (4.2) | 2 (4.2) |  | 1 (3.9) |  |  |  |  |  |  |  |  |
